# Abdominal Wall Endometriosis: Early Diagnosis of a Rare Iatrogenic Complication Following Cesarean Section

**DOI:** 10.7759/cureus.56284

**Published:** 2024-03-16

**Authors:** Anna Thanasa, Efthymia Thanasa, Ioannis-Rafail Antoniou, Gerasimos Kontogeorgis, Ektoras-Evangelos Gerokostas, Evangelos Kamaretsos, Ioannis Paraoulakis, Evangelia Simopoulou, Maria Mousia, Ioannis Thanasas

**Affiliations:** 1 Department of Health Sciences, Medical School, Aristotle University of Thessaloniki, Thessaloniki, GRC; 2 Department of Obstetrics and Gynecology, General Hospital of Trikala, Trikala, GRC; 3 Department of Pathology, General Hospital of Trikala, Trikala, GRC; 4 Department of Pathology, General Hospital Trikala, Trikala, GRC

**Keywords:** endometriosis, abdominal wall, laparotomy scar, ultrasound, computed tomography, magnetic resonance, surgical treatment, prognosis, case report

## Abstract

Abdominal wall endometriosis is a rare form of extrapelvic endometriosis, frequently diagnosed with delay in most cases. It is typically iatrogenic and primarily associated with procedures such as cesarean sections or other gynecological surgeries. In our patient, endometriosis at the laparotomy scar was diagnosed relatively early, approximately two months after the onset of symptoms, which manifested 10 months after the last cesarean section. The patient, who had an obstetric history of three cesarean sections, presented at the Gynecology outpatient clinic of the General Hospital of Trikala, complaining of pain associated with menstruation located in the lower abdomen, near the surgical scar. Based on clinical and imaging findings, abdominal wall endometriosis was suspected, leading to a decision for wide excision of the endometriotic lesion. Histological examination of the surgical specimen confirmed the diagnosis. The postoperative course was uneventful, and three months after the surgery, the patient reported complete resolution of symptoms. This case report emphasizes the importance of integrating advanced diagnostic methods alongside classic clinical findings for the accurate diagnosis of abdominal wall endometriosis. It also highlights the contribution of surgical treatment through wide excision of the endometriotic lesion, in facilitating early diagnosis, achieving cure, and minimizing the risk of disease recurrence in the future.

## Introduction

Endometriosis is a prevalent chronic gynecological condition characterized by the presence of endometrial glands or stroma in anatomical locations and organs outside the uterine cavity. Determining the precise incidence of the disease remains challenging. The estimated prevalence is approximately 5%-10% among women of reproductive age worldwide [1]. Despite heightened attention from the scientific community in recent years, the pathogenesis of endometriosis has not been fully elucidated to date [2]. Endometriosis is now recognized as a systemic disease that predominantly affects the pelvis and peritoneum. The three primary clinical variants of endometriosis include ovarian endometriomas, superficial peritoneal disease, and deep infiltrating endometriosis, which may coexist [3]. Less frequently, the disease may extend beyond the pelvis and manifest at distant sites and organs (extrapelvic endometriosis). The most common extrapelvic sites of endometriosis include the gastrointestinal tract, urinary tract, upper and lower respiratory tract, umbilicus, inguinal region, and surgical scars of the abdominal wall [4-6].

Abdominal wall endometriosis, characterized by the presence of ectopic endometrial glands or stroma within the abdominal wall, is a rare form of extrapelvic endometriosis [7]. Ectopic endometriotic tissue typically resides in the subcutaneous tissue, occasionally extending deep to the muscle fascia within the rectus abdominis muscles [8]. The precise incidence of abdominal wall endometriosis following cesarean section and other gynecological surgeries cannot be accurately determined. However, the increasing prevalence of cesarean sections performed in recent years is anticipated to substantially contribute to the rise in the incidence of abdominal wall endometriosis [9]. Abdominal wall endometriosis is commonly iatrogenic and associated with cesarean section or other open or laparoscopic gynecological surgeries involving the opening of the uterine cavity [10]. Spontaneous endometriosis of the abdominal wall, without a surgical history, represents an exceedingly rare form of extrapelvic endometriosis [11].

This case report emphasizes the rarity of abdominal wall endometriosis. The necessity of using contemporary diagnostic methods that complement the classic clinical findings in order to achieve prompt and accurate preoperative diagnosis is also highlighted. The significance of surgical treatment, particularly wide excision of the endometriotic lesion, in aiming for a cure and reducing the risk of disease recurrence, is underscored.

## Case presentation

A 29-year-old patient, with an increased body mass index (BMI = 30) and a history of three lower segment cesarean sections with Pfannenstiel incision, presented at the Gynecology outpatient clinic of the General Hospital of Trikala, approximately one year after the last cesarean section, complaining of a painful mass in the midline of the lower abdomen, located almost 1 cm below the surgical scar. The pain, which was exacerbated during menstruation, began about 10 months after the last cesarean section. The patient had been breastfeeding her newborn since birth until the time of the examination. No postoperative complications from the cesarean sections were reported based on the medical history. The patient had no history of endometriosis. Additionally, a medical disorder such as hypothyroidism was reported, which was well-regulated with appropriate medication.

**Figure 1:**
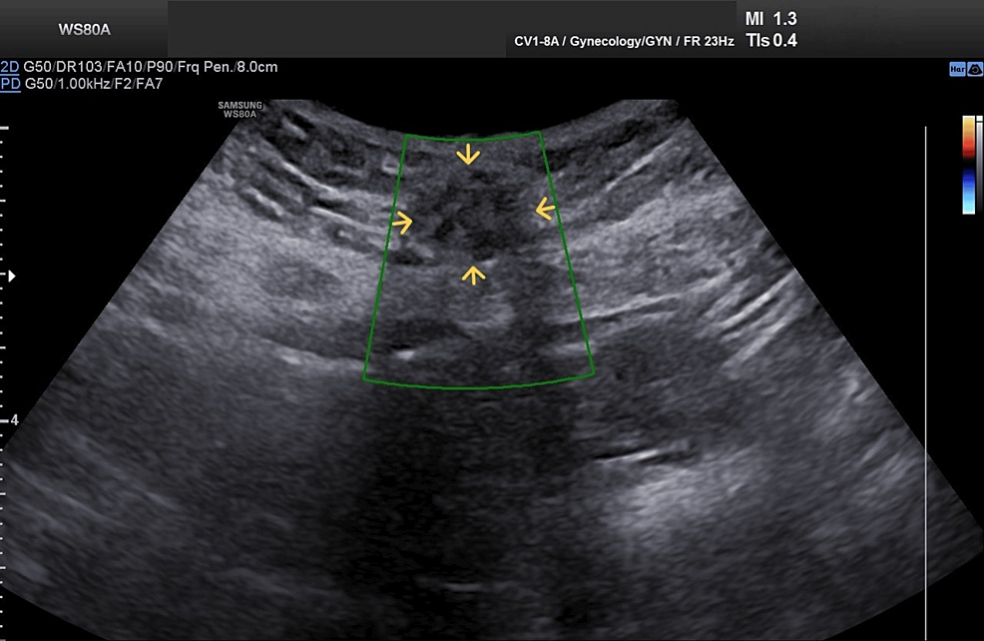
Ultrasound imaging of abdominal wall endometriosis A mixed echogenicity lesion (yellow arrows) is depicted within the subcutaneous tissue, with no evidence of vascularity on Doppler examination

On physical examination of the abdominal wall, a firm mass was noted, which elicited extreme pain upon palpation of the cesarean section scar. Transabdominal ultrasound revealed an area of mixed echogenicity within the subcutaneous tissue measuring approximately 3 cm in maximal diameter, with no evidence of vascularity on Doppler examination (Figure 1).

**Figure 2:**
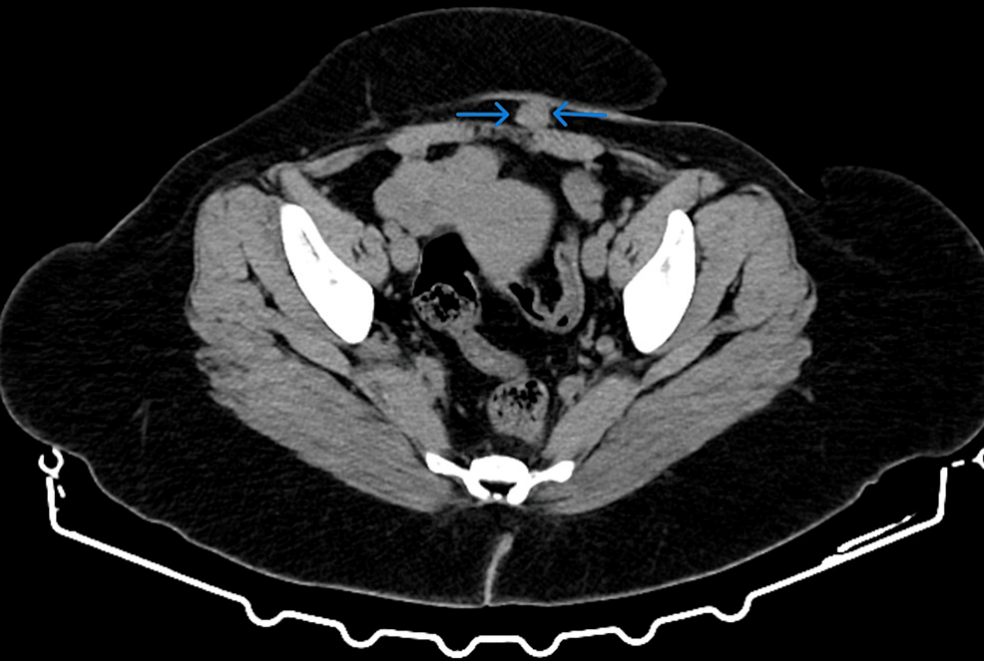
Computed tomography imaging of abdominal wall endometriosis A round lesion (blue arrows), isodense to the muscle tissue, is observed within the subcutaneous tissue and adjacent to the rectus abdominis muscle fascia, exhibiting marked and homogeneous enhancement

A computed tomography scan revealed a round, isodense to the muscle tissue mass within the subcutaneous adipose tissue and adjacent to the rectus abdominis fascia, measuring approximately 3 cm in maximal diameter, with marked and homogeneous enhancement (Figure 2).

**Figure 3:**
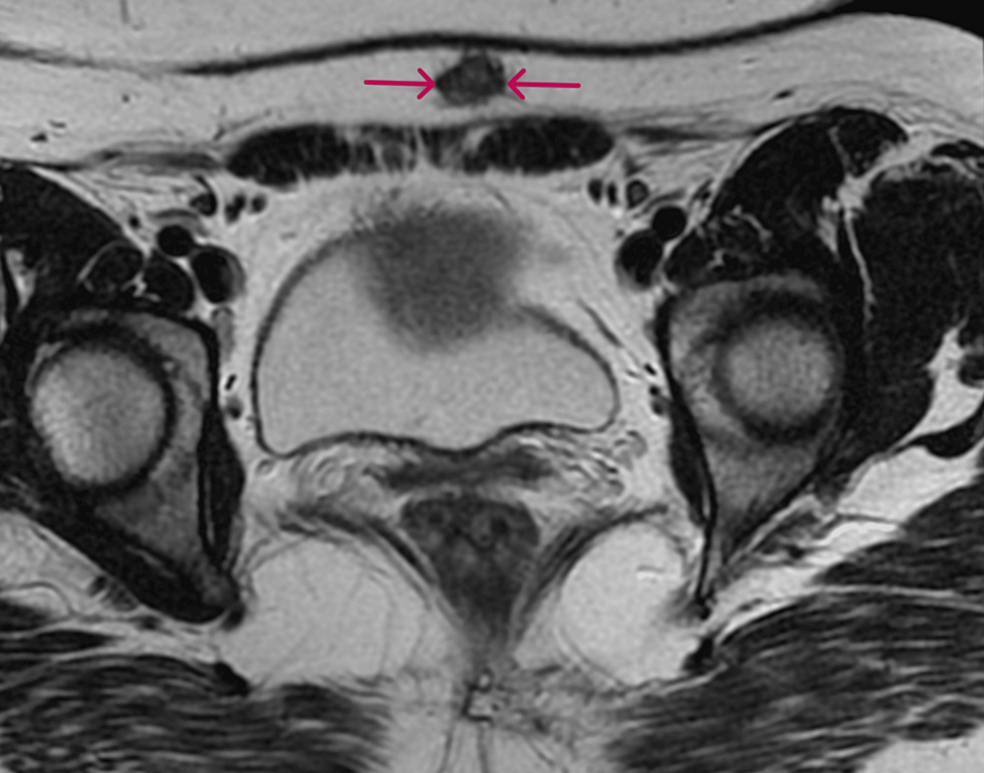
Magnetic resonance imaging of abdominal wall endometriosis Within the subcutaneous tissue, a solid spherical lesion (red arrows) is depicted, with the presence of vascularity

Magnetic resonance imaging demonstrated a solid, spherical lesion within the subcutaneous layer of the abdominal wall, with the presence of vascularity (Figure 3).

No abnormal imaging findings were observed in the uterus and adnexa. Inflammatory markers and tumor markers were within normal laboratory ranges (Table 1).

**Table 1:** Routine laboratory tests and tumor markers as part of preoperative patient screening Ht – Hematocrit, Hb – Hemoglobin, PLT – Platelets, WBC – White Blood Cells, NEUT – Neutral, APTT – Activated Partial Thromboplastin Time, INR – International Normalized Ratio, CRP – C Reactive Protein, U – Urea, Glu – Glucose, Cr – Creatinine, CEA – Carcinoembryonic Antigen, CA125 – Cancer Antigen 125, CA15-3 – Cancer Antigen 15-3, CA15-9 – Cancer Antigen 19-9

Laboratory Tests	Preoperative Values	Laboratory Reference Values
Ht	37%	37.7% – 49.7%
Hb	12.8 g/dL	11.8 – 17.8 g/dL
PLT	231x10^3^/mL	150 – 350 x10^3^/mL
WBC	7.41x40^3^/mL	4 – 10.8 x10^3^/mL
NEUT	59.6%	40% – 75%
APTT	30.3 sec	24.0 – 35.0 sec
INR	1.05	0.8 – 1.2
CRP	0.3 mg/dL	0.5 mg/dL
Glu	87 mg/dL	75 – 115 mg/dL
Cr	0.68 mg/dL	0.40 – 1.10 mg/dL
CEA	3.39 ng/mL	< 5 ng/mL
CA125	15.6 U/mL	<= 35 U/mL
CA15-3	13.8 U/mL	0.0 – 31.3 U/mL
CA15-9	12.1 U/mL	0.0 – 37 U/mL

**Figure 4:**
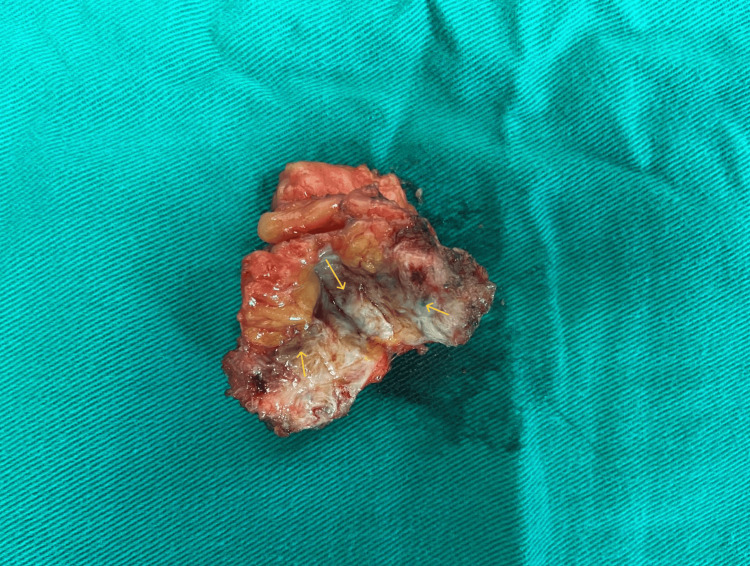
Surgical specimen of abdominal wall endometriosis Fibrous and adipose tissue, with clear ectopic endometriotic foci visible in the cross-section (yellow arrows)

Based on the clinical and imaging findings, the preoperative diagnosis of abdominal wall endometriosis was made. Wide surgical resection of the endometriotic lesion was decided. A surgical specimen measuring 5x4x4 cm, consisting of fibrous and adipose tissue (Figure 4) was excised during the procedure.

**Figure 5:**
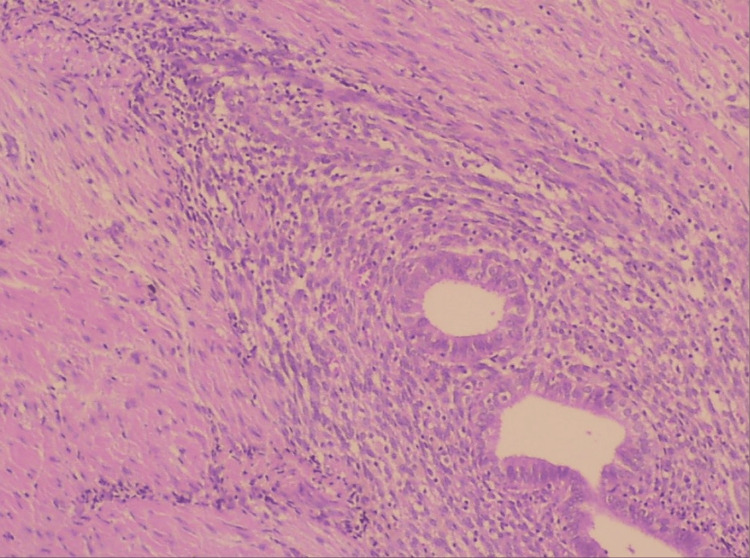
Histological image of abdominal wall endometriosis The presence of endometrial glands and stroma is evident

This specimen was found to be slightly adherent to the rectus abdominis fascia. Mesh placement was not deemed necessary by the surgical team to repair the fascial defect. The small fascial defect was completely repaired with the placement of two sutures. Histological examination of the surgical specimen confirmed the diagnosis of abdominal wall endometriosis. On microscopic examination, endometrial stroma and endometrial glands of varying size with columnar epithelium were identified (Figure 5).

The postoperative course was uneventful. The patient was discharged from the clinic on the first postoperative day following the removal of a negative pressure suction drain, which had been placed intraoperatively at the excision site of the endometriotic lesion. Three months postoperatively, the patient reported complete resolution of symptoms. Consultation was provided for regular follow-up every six months at the Gynecology outpatient clinic of the General Hospital of Trikala.

## Discussion

The diagnosis of abdominal wall endometriosis is often challenging and may be delayed [12]. Careful medical history-taking, clinical findings, and imaging modalities form the basis for an accurate and timely preoperative diagnosis of abdominal wall endometriosis. Increased body mass index, especially in association with prior abdominal surgery involving opening of the uterine cavity (as in our case), are among the main risk factors for abdominal wall endometriosis [13,14]. The time interval between surgery and the onset of symptoms is difficult to determine precisely. It is estimated to range from one to 20 years, with an average of 4.8 years [15]. In most patients, abdominal wall endometriosis presents as a palpable subcutaneous mass close to the surgical scar site, which is associated with pain and swelling that worsens during menstruation. Pain associated with menstruation is a predominant symptom in these patients. This pain, along with the presence of a palpable mass at surgical scar sites in patients with a history of caesarean section, strongly suggests the preoperative diagnosis of abdominal wall endometriosis [16]. Unlike the majority of cases, the diagnosis of abdominal wall endometriosis in our patient was not delayed. The lower abdominal pain, which first manifested 10 months after the last caesarean section and was associated with swelling of the surgical scar, was correctly not attributed to the surgery itself. Additionally, the pain was correctly not attributed to uterine contractions that can normally be induced during breastfeeding throughout the lactation period.

Besides medical history and typical clinical findings, modern imaging modalities play a crucial role in the preoperative diagnosis of abdominal wall endometriosis. Ultrasonography serves as the first-line diagnostic imaging modality for evaluating abdominal wall abnormalities [17]. Transabdominal ultrasound may reveal a well-defined hypoechoic mass within the superficial abdominal wall structures, with the possibility of detecting intralesional vascular spot Doppler ultrasound [18]. Magnetic resonance imaging is considered the gold standard imaging modality in patients with suspected abdominal wall endometriosis. Findings such as the detection of hemorrhagic foci with high signal intensity in fat-saturated T1-weighted sequences strongly support the diagnosis of abdominal wall endometriosis [19]. Computed tomography is considered valuable in differentiating abdominal wall endometriosis from other abdominal wall masses. Thus, apart from aiding in diagnosis, it is useful in decision-making regarding both possible tumor biopsy and treatment planning [20]. In our patient, the diagnosis was primarily based on medical history and clinical findings. The detection of tender swelling across the cesarean scar, especially if it recurs and is more prominent during menstruation, is indicative of the presence of ectopic endometriotic tissue within the abdominal wall. Imaging significantly aids in the differential diagnosis of endometriosis from other abdominal wall lesions. Clinical entities such as hernia, abscess, hematoma, lipoma, granulomatous tissue growth in the scar, desmoid tumor, sarcoma, and metastatic lesions should be considered in the differential diagnosis of abdominal wall endometriosis [21-23].

Furthermore, fine-needle aspiration cytology, as a simple, non-invasive diagnostic procedure, can be easily performed on superficial palpable abdominal wall lesions, enabling appropriate patient management and avoiding unnecessary diagnostic tests [24]. With fine-needle aspiration cytology, it is possible to make an accurate and timely preoperative diagnosis of abdominal wall endometriosis prior to histological examination of the surgical specimen. However, the risk of dissemination of the endometriotic lesion and recurrence of the disease must be regarded as substantial [25]. In addition to their diagnostic role, various serological markers, with cancer antigen 125 being the most important, are considered significant for proper postoperative follow-up, monitoring response to pharmacological treatment, and preventing potential recurrence or malignant transformation of the endometriotic lesion. An increase in cancer antigen 125 of more than 1,000 U/mL may indicate the presence of deeply infiltrative endometriosis [26]. In our patient, the value of cancer antigen 125 was within the normal range. The well-documented diagnosis of abdominal wall endometriosis, based on clinical and imaging findings, rendered fine needle aspiration cytology unnecessary. Hence, the risk of endometriotic foci dissemination and potential disease recurrence was avoided. In our patient, the diagnosis of abdominal wall endometriosis was confirmed through histological examination of the surgical specimen.

Wide surgical resection of the endometriotic lesion with an open approach is currently the optimal management option for abdominal wall endometriosis [9]. A laparoscopic approach may be recommended for subfascial lesions localized within the rectus abdominis muscles [7]. Primarily in such cases, as well as in patients with large endometriotic abdominal wall tumors requiring mesh placement for reconstruction, a multidisciplinary approach involving the collaboration of general surgeons is considered essential [27]. To avoid the need for mesh placement in patients with large endometriotic lesions in the abdominal wall, requiring resection of the muscle and fascia, high-intensity focused ultrasound therapy is currently being considered [28]. Intraoperatively, particularly during procedures such as caesarean sections, thorough and meticulous cleaning of the subcutaneous tissue, particularly at corner sites, is deemed essential to prevent abdominal wall endometriosis [29]. In our patient, mesh placement was not deemed necessary by the surgical team to repair the fascial defect. Two sutures were considered adequate for complete repair of the small extent of the fascial defect.

Unlike surgical treatment, pharmacological treatment as monotherapy for abdominal wall endometriosis seems unable to cure the disease. Pharmacological treatment provides only temporary symptomatic relief. The administration of contraceptive drugs, progestogens, or hormone-suppressing agents is useful for patients unwilling to undergo surgical resection of the endometriotic lesion or for those with large endometriotic masses to reduce the lesion's size preoperatively. Additionally, pharmacological treatment may be valuable for patients who underwent open excision of an endometriotic mass without clear resection margins to eliminate the increased risk of recurrence [30]. Furthermore, ultrasound-guided microwave ablation of the endometriotic lesion is an innovative therapeutic approach that shows promising results in the effective treatment of symptomatic abdominal wall endometriosis [31]. Finally, a recent case report documented in the scientific literature noted that acupuncture effectively relieves pain caused by abdominal wall endometriosis and simultaneously significantly reduces the size of the abdominal mass [32].

The prognosis of abdominal wall endometriosis is generally favorable. After surgical resection, more than 90% of patients report complete relief from symptoms [7]. In cases where the endometriotic lesion is incompletely resected from the abdominal wall, the risk of recurrence ranges between 12.5% and 28.6% [33]. Malignant transformation of abdominal wall endometriotic lesions, which has a poor prognosis, is rare. Clear cell carcinoma is the most common type, followed by endometrioid adenocarcinoma [34].

## Conclusions

Abdominal wall endometriosis is an uncommon extrapelvic manifestation of endometriosis, often linked to iatrogenic factors and frequently associated with obstetric or gynecological surgeries. In patients, especially those with a history of cesarean section, the presence of a painful palpable mass near the surgical scar, accompanied by changes associated with menstruation and a gradual increase in size, warrants consideration of abdominal wall endometriosis before attributing the clinical findings solely to the surgical procedure itself. A thorough evaluation of clinical findings, coupled with the use of modern diagnostic tests, can facilitate a timely and accurate preoperative diagnosis of abdominal wall endometriosis. Currently, wide surgical resection via an open approach is considered the preferred treatment for abdominal wall endometriosis.
